# Upstream kinases of plant SnRKs are involved in salt stress tolerance

**DOI:** 10.1111/tpj.13761

**Published:** 2017-12-02

**Authors:** Juan de Dios Barajas‐Lopez, Jose Ramon Moreno, Francisco M. Gamez‐Arjona, Jose M. Pardo, Matleena Punkkinen, Jian‐Kang Zhu, Francisco J. Quintero, Hiroaki Fujii

**Affiliations:** ^1^ Molecular Plant Biology Unit Department of Biochemistry University of Turku 20014 Turku Finland; ^2^ Instituto de Recursos Naturales y Agrobiología de Sevilla Consejo Superior de Investigaciones Cientificas 41012 Sevilla Spain; ^3^ Instituto de Bioquímica Vegetal y Fotosíntesis Consejo Superior de Investigaciones Cientificas 41092 Sevilla Spain; ^4^ Department of Horticulture and Landscape Architecture Purdue University West Lafayette IN USA; ^5^ Shanghai Center for Plant Stress Biology Shanghai Institutes for Biological Sciences Center of Excellence in Molecular Plant Sciences Chinese Academy of Sciences Shanghai 200032 China

**Keywords:** GRIKs, SnRKs, SOS2, upstream kinases, salinity, sugar, phosphorylation, stress, *Arabidopsis thaliana*

## Abstract

Sucrose non‐fermenting 1‐related protein kinases (SnRKs) are important for plant growth and stress responses. This family has three clades: SnRK1, SnRK2 and SnRK3. Although plant SnRKs are thought to be activated by upstream kinases, the overall mechanism remains obscure. Geminivirus Rep‐Interacting Kinase (GRIK)1 and GRIK2 phosphorylate SnRK1s, which are involved in sugar/energy sensing, and the *grik1‐1 grik2‐1* double mutant shows growth retardation under regular growth conditions. In this study, we established another Arabidopsis mutant line harbouring a different allele of gene *GRIK1* (*grik1‐2 grik2‐1*) that grows similarly to the wild‐type, enabling us to evaluate the function of GRIKs under stress conditions. In the *grik1‐2 grik2‐1* double mutant, phosphorylation of SnRK1.1 was reduced, but not eliminated, suggesting that the *grik1‐2* mutation is a weak allele. In addition to high sensitivity to glucose, the *grik1‐2 grik2‐1* mutant was sensitive to high salt, indicating that GRIKs are also involved in salinity signalling pathways. Salt Overly Sensitive (SOS)2, a member of the SnRK3 subfamily, is a critical mediator of the response to salinity. GRIK1 phosphorylated SOS2 *in vitro*, resulting in elevated kinase activity of SOS2. The salt tolerance of *sos2* was restored to normal levels by wild‐type SOS2, but not by a mutated form of SOS2 lacking the T168 residue phosphorylated by GRIK1. Activation of SOS2 by GRIK1 was also demonstrated in a reconstituted system in yeast. Our results indicate that GRIKs phosphorylate and activate SnRK1 and other members of the SnRK3 family, and that they play important roles in multiple signalling pathways *in vivo*.

## Significance Statement

Since plants face diverse, often coincidental stresses throughout their lifetime, they have to integrate multiple signals to organize growth, development and stress responses. This work shows that GRIKs, which are essential players in sugar signalling pathway as upstream kinases of SnRK1s, also work in salt tolerance of Arabidopsis and identified SOS2, a SnRK3, as a potential substrate of GRIKs in the salinity signalling pathway.

## Introduction

Reversible protein phosphorylation is one of the most important mechanisms by which cell signalling responds to environmental changes. Sucrose non‐fermenting 1 (SNF1)/AMP‐activated protein kinases (AMPKs) are involved in the responses to energy depletion in yeasts and mammals (Kemp *et al*., 2003; Hardie, 2004). The plant homologues of SNF1, the SNF1‐related protein kinases (SnRK), comprise three subfamilies: SnRK1, SnRK2 and SnRK3. SnRK1 proteins, including SnRK1.1 and 1.2 (also known as KIN10 and 11) of *Arabidopsis thaliana*, have the highest similarity to SNF1. As expected based on homology, SnRK1s play important roles in energy signalling (Baena‐González *et al*., 2007). On the other hand, SnRK2 and SnRK3 proteins are plant‐specific subfamilies consisting of 10 and 25 members, respectively, in Arabidopsis, and are involved in diverse signalling pathways (Hrabak *et al*., 2003; Luan, 2009; Umezawa *et al*., 2010). One of the best characterized SnRK3s (a.k.a. calcineurin B‐like proteins‐interacting protein kinases, CIPKs), SOS2 (Salt Overly Sensitive 2/SnRK3.11/CIPK24), is a Ser/Thr protein kinase acting in the SOS pathway that is required for salt tolerance (Zhu *et al*., 1998; Liu *et al*., 2000). In the SOS pathway, SOS2 activates the plasma membrane Na^+^/H^+^ antiporter SOS1 (Shi *et al*., 2000; Quan *et al*., 2007; Quintero *et al*., 2011) and vacuolar targets such as the tonoplast K^+^(Na^+^)/H^+^ exchangers NHXs (Qiu *et al*., 2004) and the H^+^/Ca^2+^ antiporter CAX1 (Cheng *et al*., 2004). SnRK3s bind to the calcium‐binding proteins, SCaBPs (SOS3‐like calcium‐binding protein, a.k.a. calcineurin B‐like/CBL), with varying degrees of specificity and combinatorial diversity (Halfter, 2000; Kim *et al*., 2000). SOS2 is activated by SOS3 or SCaBP8/CBL10 after binding to the FISL motif (a.k.a. NAF domain) of SOS2, which is thought to comprise an autoinhibitory domain, in a Ca^2+^‐dependent manner (Halfter, 2000; Quan *et al*., 2007).

In addition to interactions with binding proteins, other regulatory mechanisms of the SnRK family have been proposed. Phosphorylation in the activation‐loop (a.k.a. T‐loop) is an important regulatory mechanism of SnRK proteins (Hanks and Hunter, 1995; Cutler *et al*., 2010). In yeast, three kinases, SAK1/PAK1, TOS3 and ELM1, phosphorylate SNF1 *in vitro*, and the triple deletion of genes encoding these upstream kinases abolishes the catalytic activity of SNF1 and causes a phenotype similar to that of Δ*snf1* (Hong *et al*., 2003; Sutherland *et al*., 2003). In Arabidopsis, two kinases have been identified as homologues of yeast SAK1, TOS3 and ELM1. These proteins were named Geminivirus Rep‐Interacting Kinases (GRIK)1 and GRIK2 because their expression is induced by geminivirus infection and they bind to geminivirus replication protein AL1 (Kong and Hanley‐Bowdoin, 2002; Shen and Hanley‐Bowdoin, 2006). GRIK1 or GRIK2 (a.k.a. SnRK1 activating kinase; Hey *et al*., 2007) can functionally complement the yeast *elm1 sak1 tos3* triple mutant (Shen and Hanley‐Bowdoin, 2006). Extracts from yeast cells expressing GRIK1 or GRIK2 phosphorylate a peptide corresponding to the activation‐loop of the SnRK1 clade (Hey *et al*., 2007). In addition, recombinant GRIK1 and GRIK2 phosphorylate the activation‐loop of SnRK1.1 and 1.2, activating them *in vitro* (Shen *et al*., 2009; Crozet *et al*., 2010). A large‐scale study of double mutants suggested that the *grik1‐1 grik2‐1* double mutant was embryonic lethal (Bolle *et al*., 2013), but a recent report showed that the *grik1‐1 grik2‐1* double mutant could be rescued on sugar‐supplemented medium, albeit plants were small and did not produce seeds (Glab *et al*., 2017). Phosphorylation of SnRK1s was reduced in the double mutant (Glab *et al*., 2017). Thus, GRIK1 and GRIK2 play essential roles in sugar/energy signalling.

Resembling the activation of SnRK1 proteins, phosphorylation‐mimicking mutations in the activation‐loop make SOS2 constitutively active (Guo *et al*., 2001). Three candidate phosphorylation sites in the activation‐loop are conserved among the SnRK3 family (Gong *et al*., 2002b; Chaves‐Sanjuan *et al*., 2014). Replacement of the Ser‐156, Thr‐168 or Tyr‐175 residue in the activation‐loop of SOS2 with an Asp residue to mimic phosphorylation significantly increases the activity of the enzyme *in vitro* (Gong *et al*., 2002b). Transgenic plants overexpressing SOS2 with the Thr168→Asp mutation are more tolerant to salt (Guo *et al*., 2004), and similar effects have been observed on other SnRK3s (Gong *et al*., 2002a,c). These results strongly suggest that phosphorylation in the activation‐loop activates SOS2, raising the question of the identity of the upstream kinases involved.

To investigate the possible role of GRIK1 and GRIK2 as upstream regulators of SnRK3s, we have inspected the phenotype of Arabidopsis plants bearing mutations in genes *GRIK1* and *GRIK2*. Because the strong phenotype of the previously characterized *grik1‐1 grik2‐1* double mutant could mask the responses to environmental stress conditions, we used a different combination of mutant alleles. The *grik1‐2 grik2‐1* double mutant used here grew normally under regular conditions but was salt sensitive. We show that GRIK1 and GRIK2 phosphorylate and activate SOS2 to increase salt tolerance. Thus, GRIK1 and GRIK2 coordinate the responses to metabolic and environmental stresses in plants.

## Results

### GRIK1 and 2 affect the amount and phosphorylation state of SnRK1.1

**Figure 1:**
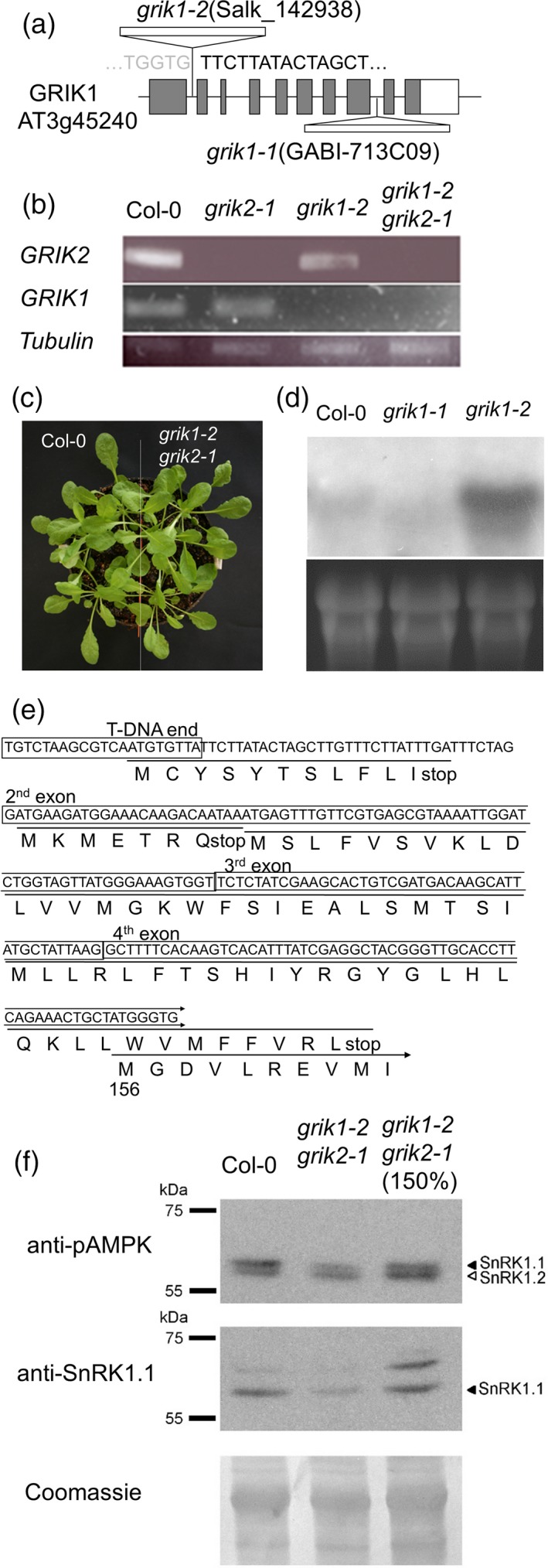
Characterization of the *grik1‐2 grik2‐1* mutant line. (a) Schematic diagram of *grik1* T‐DNA insertion lines. (b) Reverse transcriptase‐polymerase chain reaction (RT‐PCR) of full‐length *GRIK1*,* GRIK2* and *tubulin8* in the wild‐type Col‐0, *grik1‐2*,* grik2‐1* and *grik1‐2 grik2‐1*. (c) Phenotype of the wild‐type and *grik1‐2 grik2‐1* under short‐day condition for 4 weeks. (d) Northern blotting with the middle region of *GRIK1* as probe in the wild‐type, *grik1‐1* and *grik1‐2*. (e) cDNA sequence of the 5′‐end region of *grik1‐2* and potential ORFs. The longest ORF corresponded to an N‐terminal truncated GRIK1 protein starting at Met‐156 of the wild‐type protein. (f) Amount and phosphorylation status of SnRK1s in the wild‐type and *grik1‐2 grik2‐1*. Western blot with anti‐pAMPK antibody (upper panel) and anti‐SnRK1.1 antibody (middle panel). Coomassie staining was used to show protein amounts (lower panel). A greater amount of proteins from *grik1‐2 grik2‐1* (150%) was also loaded to compare the phosphorylation ratio in equivalent amounts of SnRK1.1.

To investigate the function of GRIK1 and GRIK2 *in vivo*, we analysed T‐DNA insertion lines in which either the *GRIK1* or *GRIK2* genes were disrupted (Salk_142938 for *grik1* and Salk_015230 for *grik2*; Figure 1a). For this study, we will denote these mutant alleles as *grik2‐1* as this is the only GRIK2 knockout line characterized so far, and *grik1‐2* because this is a *grik1* allele different to the GABI‐713C09 mutant reported previously (Bolle *et al*., 2013; Glab *et al*., 2017). Because we observed no significant differences between the wild‐type, *grik1‐2* and *grik2‐1* plants, we crossed the two mutant lines and identified a *grik1‐2 grik2‐1* double mutant in the F2 generation. We verified that the mutant lines *grik1‐2* and *grik2‐1* used in this study harbour T‐DNA insertions in the 1st intron and 10th exon, respectively, and confirmed by reverse transcriptase‐polymerase chain reaction (RT‐PCR) that no full‐length mRNA for either *GRIK1* or *GRIK2* is expressed in the double mutant (Figure 1b). A large‐scale study of double mutants of paralogous genes suggested that the *grik1‐1 grik2‐1* double mutant was embryonic lethal (Bolle *et al*., 2013). Recently, Glab *et al*. (2017) have shown that the *grik1‐1 grik2‐1* mutant could be rescued by sugar supplementation, yet the mature plant was stunted and sterile. The *grik1‐2 grik2‐1* double mutant used here grew and reproduced normally under our growth‐room conditions (Figure 1c). Thus, it was possible that *grik1‐2* is a weak allele and not a complete loss‐of‐function. To investigate the possibility that the T‐DNA insertion in the 1st intron yielded a truncated form of GRIK1 in the *grik1‐2* mutant, Northern blotting was performed using as probe the middle region of *GRIK1*, downstream of the T‐DNA insertion. In the *grik1‐2* mutant, a hybridizing band stronger than the endogenous *GRIK1* transcript in the wild‐type was detected, while no band was detected with similar size to the wild‐type in the *grik1‐1* mutant (Figure 1d). Sequencing of the RT‐PCR amplicon pertaining to the *grik1‐2* allele revealed that there was an mRNA containing the T‐DNA left border fused to the 1st intron and the second exon of *GRIK1* in the 5′‐end region of *grik1‐2* mRNA. Because the 2nd and 3rd introns were correctly spliced out, the remaining mRNA may have the same sequence as the wild‐type in the downstream 3′‐end region. While several short ORFs could possibly encode peptides 7‐, 11‐ and 59‐residues long, the longest ORF in the *grik1‐2* transcript encoded an N‐terminal truncated GRIK1 protein starting at Met‐156 in the wild‐type protein (Figure 1e). These results and the phenotype of the *grik1‐2* mutant suggest that this truncated GRIK1 expressed in the *grik1‐2* mutant could provide residual GRIK1 function. Importantly, the robust growth of the *grik1‐2 grik2‐1* double mutant allowed the inspection of stress‐related responses, which could be separated from the role of GRIKs in plant development.

SnRK1s are the primary targets of GRIKs in Arabidopsis (Shen *et al*., 2009). In the *grik1‐1 grik2‐1* mutant the phosphorylation in the activation‐loop of SnRK1.1 was almost eliminated (Glab *et al*., 2017). To evaluate the phosphorylation rate in the *grik1‐2 grik2‐1* mutant, we used an antibody against phosphorylated AMPK protein that recognizes the Arabidopsis SnRK1 proteins (Baena‐González *et al*., 2007; Cho *et al*., 2016). Results showed that the amounts of phosphorylated SnRK1.1 and SnRK1.2 were lower in the *grik1‐2 grik2‐1* mutant compared with wild‐type on Murashige and Skoog medium (MS) plates with 1% sucrose, although phosphorylation was not entirely eliminated (Figure 1f). Western blotting with the anti‐SnRK1.1 antibody showed that the total amount of SnRK1.1 was also reduced in the *grik1‐2 grik2‐1* double mutant (Figure 1f). These results suggest that the phosphorylation and amount of SnRK1 proteins are reduced in the *grik1‐2 grik2‐1* mutant.

### The *grik1‐2 grik2‐1* double mutant is sensitive to glucose during post‐germination growth

Next, we examined the phenotype of the *grik1‐2 grik2‐1* double mutant under low‐energy conditions, in which SnRK1s play important roles (Baena‐González *et al*., 2007). Four‐week‐old plants were kept in the dark or submerged in water for 60 h, conditions in which mutants with altered SnRK1.1 activity manifested phenotypic differences with the wild‐type (Cho *et al*., 2016). We detected no significant growth difference between the wild‐type and *grik1‐2 grik2‐1* double mutant in the dark (Figure S1a) or under submergence (Figure S1b), suggesting that the residual phosphorylation of SnRK1s is sufficient to mediate physiologically important functions under the tested conditions. By contrast, the *grik1‐2 grik2‐1* double mutant had a glucose‐sensitive phenotype. On MS plates with 1% sucrose, *grik1‐2 grik2‐1* showed post‐germination growth similar to the wild‐type. On the other hand, growth of the double mutant was arrested before greening on MS plates with 1% sucrose and supplemented with 3% glucose (Figure S1c). By contrast, on plates with 3% additional sucrose (total 4%), we observed no difference in greening between the wild‐type and the double mutant, although the wild‐type tended to grow slightly faster (Figure S1c). On 3% sorbitol plates, in which the osmolarity was identical to that of the glucose‐ and sucrose‐supplemented plates, we observed no difference in growth between the wild‐type and the double mutant (Figure S1c). These results indicate that GRIK1 and GRIK2 specifically function in high‐glucose conditions, which is consistent with the reported phenotype of the *grik1‐1 grik2‐1* mutant (Glab *et al*., 2017).

### The *grik1‐2 grik2‐1* double mutant is sensitive to high concentrations of NaCl

**Figure 2:**
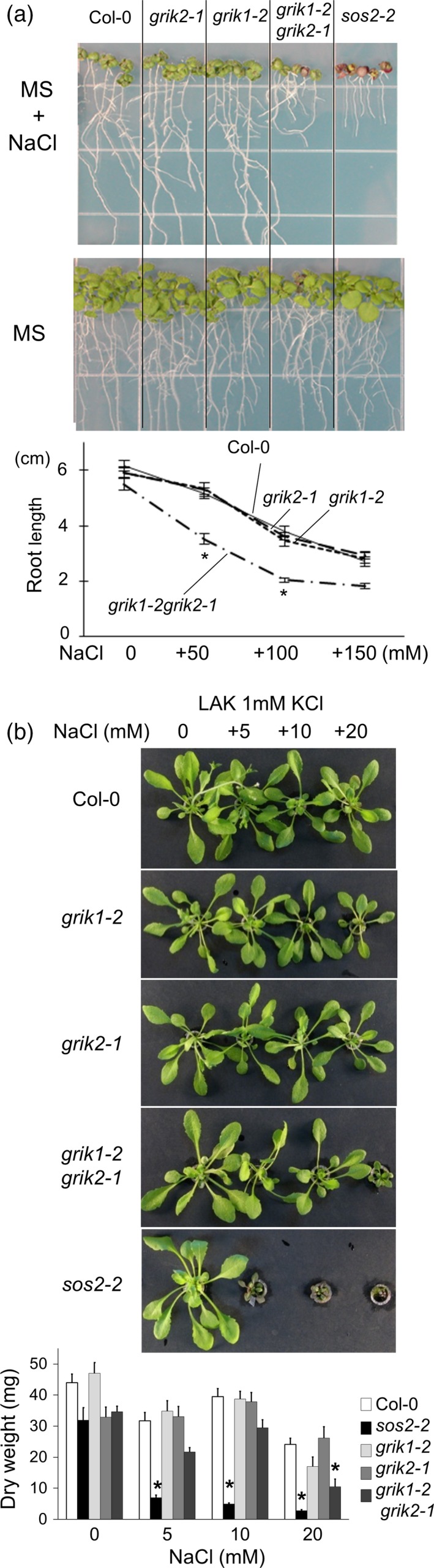
Salt‐sensitive phenotype of *grik1‐2 grik2‐1* double mutant. (a) Seedlings of the wild‐type (Col‐0), *grik1‐2*,* grik2‐1*,* grik1‐2 grik2‐1* and *sos2‐2* mutants grown on Murashige and Skoog (MS) agar plate were transferred to fresh plates with or without 100 mm NaCl. Photographs were taken 12 days after transfer. The line graph represents primary root length of seedlings 12 days after transfer to MS agar plates with the indicated concentration of NaCl (mean ± SE, *n* = 20). Asterisks indicate significant differences from wild‐type (*P *< 0.05, in one‐way anova followed by Tukey's multiple comparison test). (b) Hydroponic culture of Col‐0, *grik1‐2*,* grik2‐1*,* grik1‐2 grik2‐1* and *sos2‐2* mutants in LAK medium supplemented with NaCl as indicated. Plants were grown for 4 weeks. The bar graph represents the dry weight of plants (mean ± SE, *n* = 7) at the end of the experiment. Asterisks indicate significant differences from wild‐type (*P* < 0.01, in one‐way anova followed by Tukey's multiple comparison test).

Next, we assessed the sensitivity of the double mutant to environmental stresses. When similarly‐sized 3–4‐day‐old seedlings were transferred to MS agar plates supplemented with 50 mm or 100 mm NaCl, the roots of the double mutant were shorter than those of the wild‐type, *grik1‐2* or *grik2‐1* (Figure 2a); on regular MS plates, the double mutant had slightly shorter roots. The *grik1‐2 grik2‐1* double mutant was not as sensitive to NaCl as the *sos2‐2* mutant, which could not survive on 100 mm NaCl plates (Figure 2a). On the other hand, when seedlings were transferred to MS plates supplemented with 200 or 300 mm mannitol, we detected no significant differences in root length among any of the strains tested, in keeping with the similar sensitivity of the double mutant and wild‐type plants to 3% sorbitol (165 mm; Figure S1d). These results indicate that GRIK1 and GRIK2 are instrumental in mounting tolerance to the ionic component of salinity and are likely to function in concert with the SOS pathway. Hence, we also evaluated the contribution of GRIK1 and GRIK2 to the salt tolerance of Arabidopsis in hydroponic culture with LAK medium, which maximises ionic‐sensitive phenotypes due to the enhanced sodium uptake linked to evapotranspiration (Barragan *et al*., 2012). The *grik1‐2 grik2‐1* double mutant had a significant salt‐sensitive phenotype at 20 mm NaCl, but was still less sensitive than the *sos2‐2* mutant (Figure 2b). Together, these results indicate that GRIK1 and GRIK2 play redundant yet important roles in the sodium tolerance of Arabidopsis.

### GRIK1 phosphorylates Thr168 in the activation‐loop of SOS2

**Figure 3:**
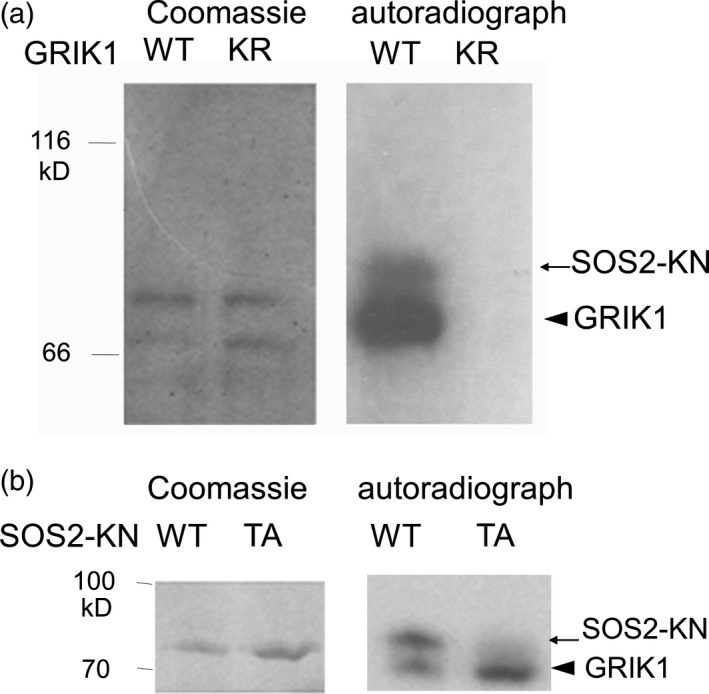
*In vitro* phosphorylation of SOS2 by GRIK1. (a) A kinase‐dead SOS2 fused to GST (SOS2‐KN, arrow) was incubated with GST‐fused GRIK1 or the kinase‐dead (K137R) of GRIK1 (GRIK1‐KR) also fused to GST (arrowhead). Proteins were separated in sodium dodecyl sulphate–polyacrylamide gel electrophoresis (SDS–PAGE) followed by Coomassie staining (left) and autoradiograph (right). (b) GST‐fused mutant T168A of SOS2 was incubated with GST‐fused GRIK1. Proteins were separated in SDS–PAGE followed by Coomassie staining (left) and autoradiograph (right).

SOS2, which belongs to the SnRK3 subfamily and plays an important role in salt tolerance, is phylogenetically related to SnRK1s. Hence, we investigated whether GRIKs could also phosphorylate SOS2. For these experiments, we produced glutathione S‐transferase (GST)‐fused GRIK1 recombinant proteins in *Escherichia coli*. A kinase‐dead type mutant protein of GRIK1 (GRIK1‐KR), in which the ATP‐binding residue Lys137 was changed to Arg, was also produced as a negative control. *In vitro* kinase assays revealed that GRIK1 had autophosphorylation activity, whereas GRIK1‐KR did not (Figure 3a). A kinase‐dead type of SOS2, in which Lys40 was mutated to Gln (SOS2‐KN; Gong *et al*., 2002b), was phosphorylated by GRIK1, but not by GRIK1‐KR (Figures 3a and Figure S2). These results indicate that GRIK1 phosphorylates SOS2 *in vitro*.

To identify the GRIK1 phosphorylation site in SOS2, we introduced mutations in the activation‐loop at the three candidate sites (Ser‐156, Thr‐168 and Tyr‐175), because they are fully conserved in all the members of the Arabidopsis SnRK3 subfamily, and replacement of these residues with Asp to mimic phosphorylation significantly increased the activity of SOS2 *in vitro* (Gong *et al*., 2002b; Chaves‐Sanjuan *et al*., 2014). We generated SOS2 mutants, in which one or all three of the putative phosphorylated residues were changed to Ala (single mutants SOS2‐S156A, SOS2‐T168A and SOS2‐Y175A; and triple mutant SOS2‐AAA), and used them as GRIK1 substrates in an *in vitro* kinase assay. These mutations of SOS2 were combined with the K40N mutation to abrogate auto‐phosphorylation. Phosphorylation of SOS2‐T168A and SOS2‐AAA by GRIK1 was not detected, whereas SOS2‐S156A and SOS2‐Y175A were still phosphorylated by GRIK1 (Figures 3b and S2). These results suggest that GRIK1 phosphorylates Thr168 in the activation‐loop of SOS2 *in vitro*.

### The T168A mutant of SOS2 cannot rescue the salt‐sensitive phenotype of *sos2‐2*

**Figure 4:**
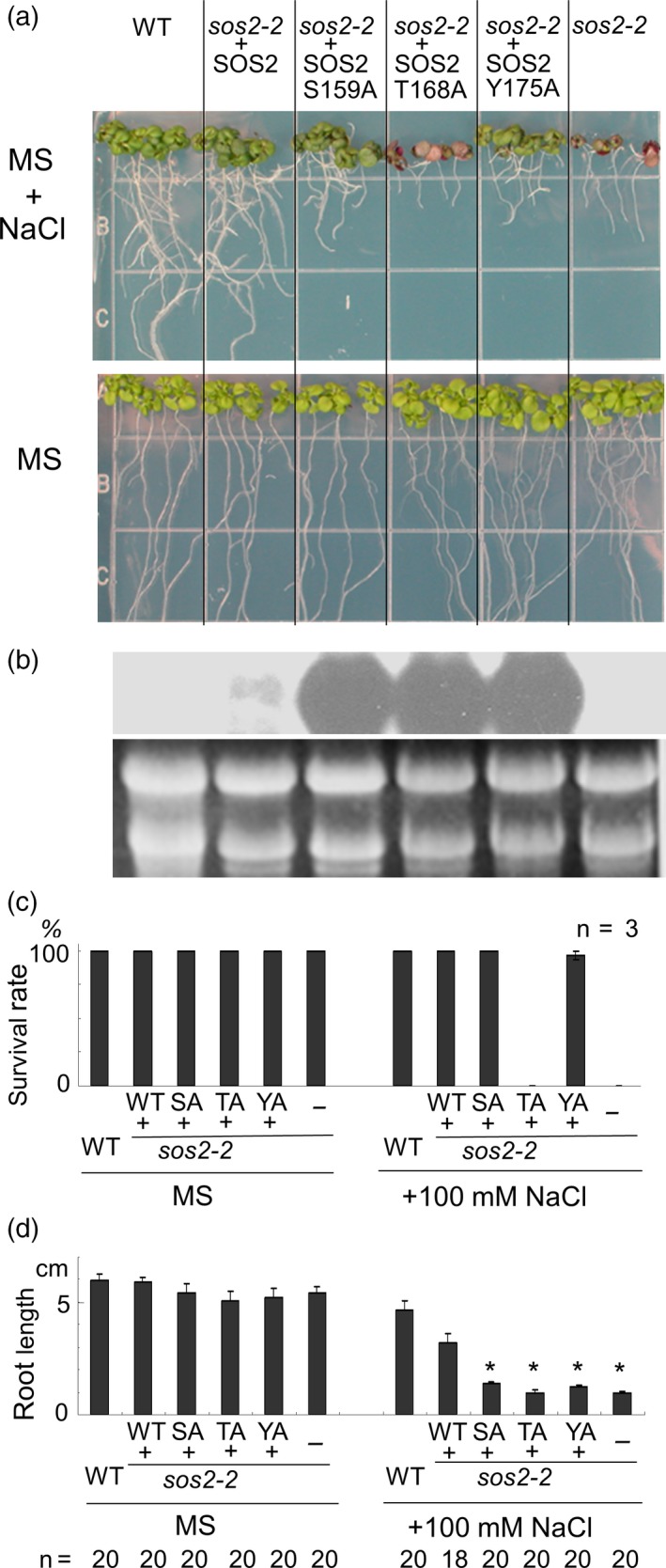
Expression of SOS2 mutants S159A (SA) and Y175A (YA), but not T168A (TA), partially rescued the *sos2‐2* under salt stress condition. (a) Seedlings grown on Murashige and Skoog (MS) agar medium were transferred to fresh plates with or without 100 mm NaCl. Photographs were taken 12 days after transfer. The lines presenting the best resistance to the NaCl plates were selected for this test. (b) Northern blot for *SOS2* transcript was performed with total RNA extracted from wild‐type (WT), *sos2‐2* transgenic plants expressing the S159A (SA), T168A (TA) and Y175A (YA) mutant forms of SOS2, and non‐transformed *sos2‐2*. Total RNA was purified 12 h after transfer to MS agar plates with 100 mm NaCl. rRNA (ethidium bromide stained) was used as a loading control. (c) Survival rates of the seedlings on MS agar plates with or without 100 mm NaCl. The experiment was repeated three times, and mean values (± SE) are shown. (d) Primary root length of seedlings 12 days after transfer to MS agar plates with or without 100 mm NaCl (mean ± SE). Asterisks indicate significant differences from *sos2‐2* transformed with the wild‐type *SOS2* (*P *< 0.05, in one‐way anova followed by Tukey's multiple comparison test).

We next examined whether the phosphorylation site in the activation‐loop is essential for the activity of SOS2 under salt stress *in vivo*. For this purpose, phosphorylation site‐mutated forms of SOS2 were expressed in the *sos2‐2* background under the control of the 35S promoter of Cauliflower Mosaic Virus (35S promoter). Transgenic plants expressing wild‐type SOS2 in the *sos2‐2* background could grow well under high‐salt conditions (Figure 4). Transgenic plants expressing SOS2‐S156A or SOS2‐Y175A in the *sos2‐2* background could survive on 100 mm NaCl plates, albeit their roots were shorter than those of the plants expressing wild‐type SOS2. The lines presenting the best resistance to the NaCl plates among more than 10 independent lines for each transgene were selected for these experiments in Figure 4. On the other hand, all the 12 lines tested of T2 transgenic plants expressing SOS2‐T168A in the *sos2‐2* background died on the 100 mm NaCl plates (Figure 4). Northern blot analysis revealed that transcripts of SOS2‐S156A, SOS2‐T168A and SOS2‐Y175A in each transgenic line were substantially more abundant than those of wild‐type *SOS2* in the transgenic line expressing wild‐type or endogenous *SOS2* (Figure 4b). The reason for this disproportionate accumulation of SOS2‐S156A, SOS2‐T168A and SOS2‐Y175A transcripts is unclear, but could be due to the known upregulation of the *SOS2* transcript by salt stress, and that transgenic plants expressing inactive SOS2 mutants would suffer from acute salinity stress compared with the wild‐type and *sos2‐2* transgenic plants complemented by the wild‐type *SOS2* gene. In any case, transgenic lines expressing the SOS2‐S156A, SOS2‐T168A and SOS2‐Y175A transcripts at levels commensurate with the wild‐type *SOS2* transgene showed similar complementation results (Figure S3), demonstrating that differences in transgene expression were not the reason for root growth retardation in plants expressing SOS2‐S156A or SOS2‐Y175A. Together, these results indicate that Ser156, Thr168 and Tyr175 of SOS2 are involved in the function of SOS2 under salt stress, and that Thr168 in particular is essential for survival under salt stress.

### *In vitro* activation of SOS2 by GRIK1

**Figure 5:**
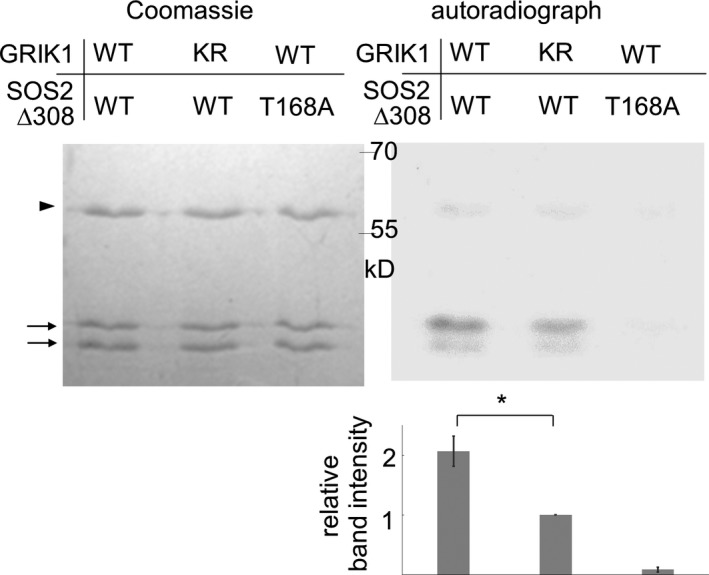
*In vitro* activation of SOS2‐∆308 by GRIK1. The GST‐fused SOS3‐independent form of SOS2 (SOS2‐∆308, arrowhead), or the combined T168A mutation in SOS2‐∆308 were incubated with GST‐fused GRIK1 or GST‐fused kinase‐dead (K137R) version of GRIK1 (GRIK1‐KR). The SOS1 C‐terminal fragment (SOS1‐CT, arrows) was added as a phosphorylation substrate of SOS2. Proteins were separated in sodium dodecyl sulphate–polyacrylamide gel electrophoresis (SDS–PAGE) followed by Coomassie staining (left) and autoradiograph (right). Note that the small amount of GRIK1 used was enough to achieve the activation of SOS2, even though GRIK1 bands were not observed. The relative band intensity of SOS1‐CT bands was normalized to the signal from the SOS2‐∆308WT/GRIK1‐KR lane (mean ± SE, *n* = 6). Asterisks indicate significant differences (*P *< 0.05, binomial test).

Next, we asked whether phosphorylation by GRIK1 activates SOS2 *in vitro*. Deletion of the C‐terminal 138 amino acids of SOS2 (SOS2‐∆308) removes the autoinhibitory domain of SOS2, yielding a SOS3‐independent form of the kinase (Guo *et al*., 2001). The T168A‐mutated form of SOS2‐∆308 (SOS2‐∆308/T168A) was used as a negative control. The GST‐fused C‐terminal 148 amino acids of SOS1 (SOS1‐CT; Fujii and Zhu, 2009) were used as a substrate for SOS2. SOS1‐CT was phosphorylated by SOS2‐∆308 to a greater degree in the presence of GRIK1 than in the presence of GRIK1‐KR (Figures 5 and S2). Little phosphorylation of SOS1 was observed when GRIK1 was combined with the SOS2‐∆308/T168A protein. These results indicate that phosphorylation of Thr168 by GRIK1 can activate SOS2 *in vitro*.

**Figure 6:**
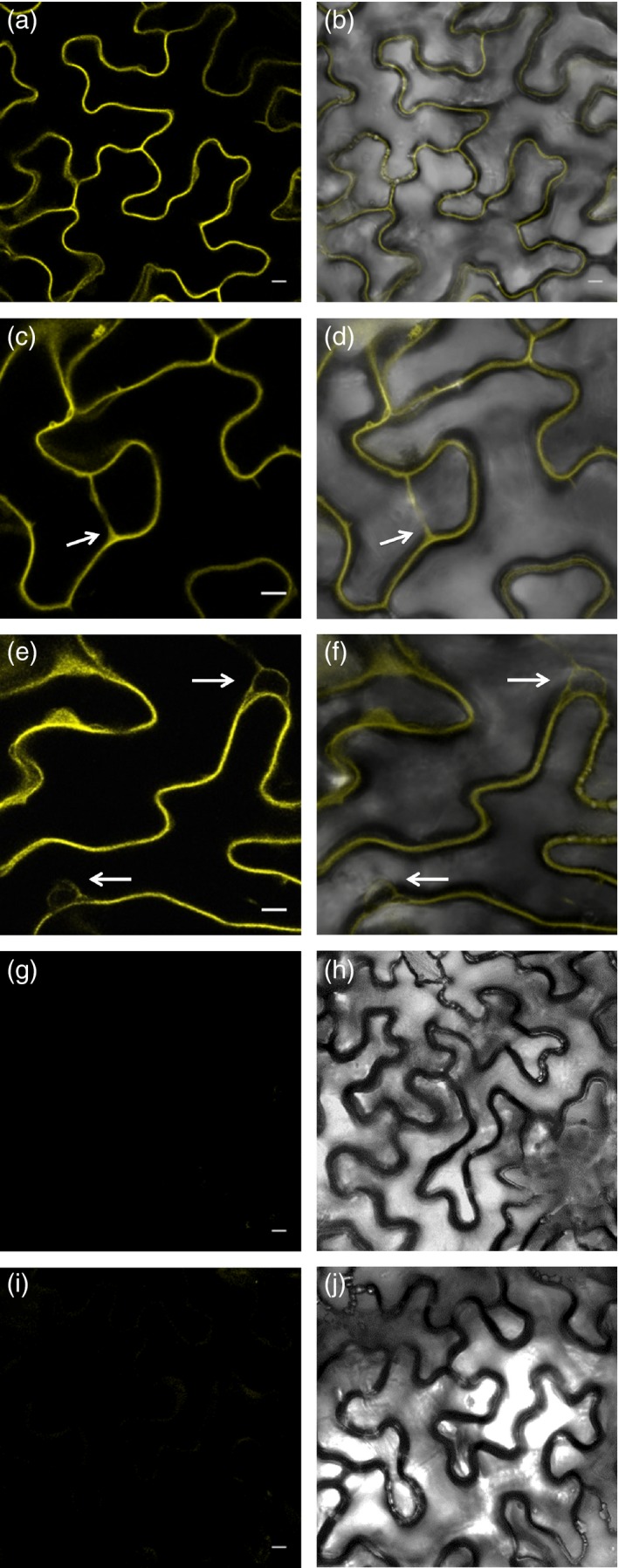
SOS2‐GRIK1 interaction visualized by bimolecular fluorescence complementation (BiFC). Panels from (a) to (f) show several images of reconstituted BiFC by GRIK1‐SOS2 interaction (left: fluorescence images; right: overlay with transmitted light). Arrows indicate the presence of a transvacuolar strand in (c) and (d), and nuclei in (e) and (f). Panels (g) and (h) show a negative control with GRIK1 in pSPYCE(M) and the empty vector pSPYNE(R)173. Panels (i) and (j) show co‐transformation with SOS2 in pSPYNE(R)173 and the empty vector pSPYCE(M). Scale bar: 10 μm.

To confirm the physical interaction of GRIK1 and SOS2 *in planta*, we conducted bimolecular fluorescence complementation (BiFC) experiments in *Nicotiana benthamiana*. The results demonstrated that SOS2 and GRIK1 interact at least in the cytosol (Figure 6). BiFC fluorescence was visible in the cytoplasmic rims around nuclei, in cytosol‐filled transvacuolar strands, and in cytoplasmic pockets. Although SOS2 shows a nucleo‐cytoplasmic distribution when expressed alone (Kim *et al*., 2007), no GRIK1–SOS2 complex was detected inside the nuclei.

### GRIK1 activates SOS2 in yeast

The yeast SNF1 kinase is essential for carbon utilization, as evidenced by the inability of yeast to grow in non‐fermentable carbon sources if SNF1 is inactive. To test the ability of GRIKs to activate SNF1, Arabidopsis GRIK1 was expressed in the yeast strain YPDahl55 (*sak1Δ*::*KanMX elm1Δ*::*KanMX tos3Δ*::*TRP1*), which lacks all three upstream regulatory kinases of SNF1 (Ye *et al*., 2008). Restoration of growth in media with the non‐fermentable carbon sources glycerol and ethanol by the wild‐type GRIK1 but not by the dead‐kinase mutant bearing the mutation K137R demonstrated that GRIK1 was fully active in the yeast cell (Figure S4).

**Figure 7:**
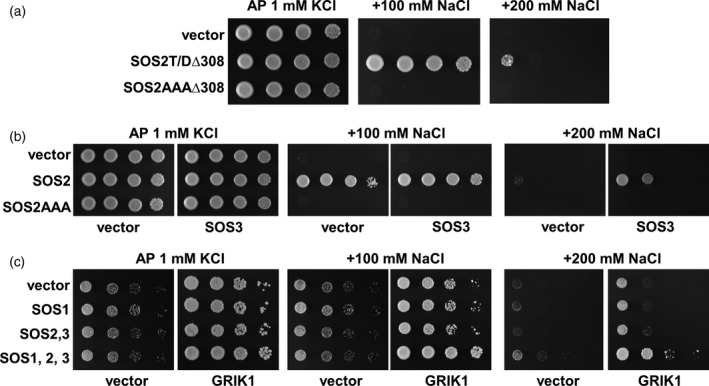
Activation of SOS2 by GRIK1 in yeast. (a) The constitutively active form of SOS2 lacking the C‐terminal autoinhibitory domain and with either the phosphomimic T168D mutation in the activation‐loop (SOS2‐T/D∆308) or with the three putative phosphorylation sites by GRIK kinases (S156/T168/Y175) converted to alanine residues (SOS2‐AAA∆308) were expressed in the yeast strain YP890 bearing mutations *ena1‐4 nha1 nhx1* and expressing SOS1 from a chromosomal integration. The salt sensitivity of all transformants was analysed by spotting decimal dilutions of starting cultures in AP plates supplemented with the indicated amounts of NaCl. Growth in NaCl‐supplemented media reported the activation of SOS1 by SOS2. (b) The full‐length SOS2 protein and the triple mutant S156A/T168A/Y175A (SOS2‐AAA) were expressed in the yeast strain YP890 harbouring a chromosomal integration of SOS1. When indicated, SOS3 was also co‐expressed. The salt tolerance of the transformants was analysed in AP medium with increasing concentration of NaCl as described above. Failure to convey salt tolerance indicated that SOS2AAA is unable to form a productive complex with SOS3 to activate SOS1. (c) SOS1, SOS2 and SOS3 were expressed, in various combinations as indicated, in strain Δ3K4E, which lacks the three SNF1‐activating kinases SAK1, ELM1 and TOS3 in addition to the Na^+^ pumps ENA1‐4. When indicated, GRIK1 was also co‐expressed to test for the ability to complement the *sak1 elm1 tos3* mutations with regard to SOS2 activation. Results indicated that only the full complement of GRIK1, SOS2, SOS3 and SOS1 was able to restore salt tolerance.

The SOS pathway, comprising the Na/H exchanger SOS1, SOS2 and SOS3, can be reconstituted in yeast strains that lack all major sodium efflux transporters and are exceedingly sensitive to sodic stress (Quintero *et al*., 2002, 2011). We used this system to analyse the *in vivo* activation of SOS2 by GRIK1. For this study, we used strain YP890, a derivative of AXT3K (∆*ena1*::*HIS3*::*ena4*,* nha1*::*LEU2*,* nhx1*::*KanMX*; Quintero *et al*., 2002), in which a *PGK1*_*prom*_:*SOS1*:*CYC1*_*ter*_ expression cassette was inserted chromosomally to provide moderate and constitutive expression of the SOS1 protein (Guo *et al*., 2004). This strain expresses the endogenous SNF1‐upstream kinases SAK1/PAK1, ELM1 and TOS3, which are the fungal homologues of GRIKs (Shen and Hanley‐Bowdoin, 2006). For reconstitution of the GRIK1/SOS2/SOS1 phosphorylation cascade, we first tested the non‐phosphorylatable mutant SOS2‐AAA in strain YP890. SOS2‐AAA was unable to activate SOS1 in this system, irrespective of the removal of the autoinhibitory domain of SOS2 (SOS2‐AAAΔ308; Figure 7a) or the co‐expression of SOS3 (Figure 7b). These results indicate that, in contrast to wild‐type SOS2, the SOS2‐AAA mutant cannot be activated by endogenous upstream kinases.

Next, we generated strain Δ3K4E, in which the genes encoding the three fungal upstream kinases SAK1/PAK1, ELM1 and TOS3, and the ENA1‐4 sodium pumps that are the major determinants of Na^+^ tolerance in *Saccharomyces cerevisiae*, had been deleted. Transformation of Δ3K4E cells with the core components of the Arabidopsis SOS pathway (SOS1, SOS2 and SOS3) failed to complement the salt‐sensitive phenotype (Figure 7c). Expression of GRIK1 in Δ3K4E cells improved growth in glucose‐supplemented AP medium due to the complementation of *sak1 elm1 tos3* mutations. At low‐NaCl concentrations (100 mm NaCl), the residual salt tolerance imparted by the full complement of SOS proteins was identical to that conferred by SOS1 alone, indicating that the SOS2–SOS3 regulatory module was essentially inactive in Δ3K4E (Figure 7c). However, co‐expression of GRIK1 increased the capacity of the SOS proteins to confer salt‐tolerance to Δ3K4E cells. These results demonstrate that GRIK1 can activate SOS2 in yeast.

## Discussion

In this study, we successfully produced a *grik1‐2 grik2‐1* double mutant with near wild‐type vegetative growth. Previous data had indicated that the *grik1‐1 grik2‐1* double mutant was lethal before germination (Bolle *et al*., 2013) or needed supplementation of sugar to grow beyond the cotyledon‐stage (Glab *et al*., 2017). Whereas the *grik2‐1* mutant line used here (Salk_015230) is identical to that of previous reports, the mutant lines harbouring T‐DNA insertions in *GRIK1* were different. Previous studies used the GABI line 713C09 with the T‐DNA insertion in the 8th intron, and we have used line Salk_142938 bearing the T‐DNA insertion at the 1st intron (Figure 1a). Northern blotting and RT‐PCR data (Figure 1d and e) suggest that in *grik1‐2* the T‐DNA insertion disrupts the full‐length *GRIK1* transcript but allows the expression of a mRNA encoding a truncated form of GRIK1 starting at methionine 156. Our results showing that phosphorylation of SnRK1s at the activation‐loop was reduced but not eliminated in the *grik1‐2 grik2‐1* double mutant (Figure 1f) suggest that the putative protein encoded by the *grik1‐2* allele retains some level of activity. The remaining phosphorylation of SnRK1s may be also sufficient to maintain SnRK1‐mediated pathways, as the *grik1‐2 grik2‐1* double mutant did not exhibit any defects under dark or submergence conditions (Figure S1a and b), in which SnRK1s play important roles (Baena‐González *et al*., 2007; Cho *et al*., 2016). On the other hand, the *grik1‐2 grik2‐1* double mutant was sensitive to high glucose (Figure S1c) as previously shown for the *grik1‐1 grik2‐1* double mutant (Glab *et al*., 2017), indicating that the truncated protein encoded by the *grik1‐2* allele cannot fully replace the intact GRIK1. Lack of GRIKs might have multiple effects on SnRK1‐mediated pathways, including a decrease in the amount of SnRK1.1 in the *grik1‐2 grik2‐1* double mutant (Figure 1f). In the *grik1‐1 grik2‐1* double mutant, the amount of SnRK1.1 was similar to that of the wild‐type (Glab *et al*., 2017). Because SnRK1.1 degradation is strictly dependent on its activity and inactive SnRK1.1 variants are disproportionally stable (Crozet *et al*., 2016), it is likely that the synthesis of SnRK1.1 is somehow compromised in both double mutants compared with the wild‐type, whereas higher degradation of SnRK1.1 would occur in *grik1‐2 grik2‐1* compared with the *grik1‐1 grik2‐1* mutant due to the residual activity of SnRK1.1 in the *grik1‐2* background.

The *grik1‐2 grik2‐1* double mutant was sensitive to high NaCl (Figure 2). The effects on salt sensitivity indicated that GRIKs play important roles in several signalling pathways, and suggested that they might have substrates other than SnRK1s. Shen *et al*. (2009) concluded that GRIK1 and GRIK2 do not phosphorylate SOS2. However, their *in vitro* kinase assay detected bands possibly corresponding to phosphorylated SOS2, but none corresponding to phosphorylated SnRK2.4. In our experiments, phosphorylation of SOS2 by GRIK1 was strong enough to be detectable (Figure 3a). Based on the observation that phosphorylation intensity of the mutated form of SOS2 (T168A, T169A) was similar to that of the wild‐type SOS2, Shen *et al*. (2009) also concluded that GRIK1 and GRIK2 could not phosphorylate the activation‐loop of SOS2. We observed that the phosphorylated SOS2 band disappeared in SOS2‐T168A and SOS2‐AAA (Figures 3b and S2c), indicating that GRIK1 indeed phosphorylates SOS2 at T168. The reason for the discrepancy between our results and those of Shen *et al*. (2009) remains unclear. Under their conditions, SOS2 (T168A, T169A) may have been phosphorylated on other sites to a sufficient degree to mask the reduction of phosphorylation on T168/T169; alternatively, the difference may be due to unintended effects of the fused tags. The T168 residue of SOS2 is conserved among SnRK3s (Gong *et al*., 2002b), whereas this residue is a Ser in SnRK2s (with the exception of SnRK2.8). This suggests that SnRK3s, but not SnRK2s, may be targeted by GRIKs. In our study, GRIK1 phosphorylated and activated SOS2‐∆308 *in vitro* (Figure 4). GRIK1 and SOS2 physically interact in *N. benthamiana* (Figure 6). In addition, GRIK1 could activate the SOS pathway in a yeast‐reconstituted system (Figure 7). Taken together, our results indicate that GRIK1 has the ability to phosphorylate the activation‐loop of SOS2 and to activate this kinase, suggesting that the roles of GRIKs are not limited to upstream regulation of SnRK1s.

**Figure 8:**
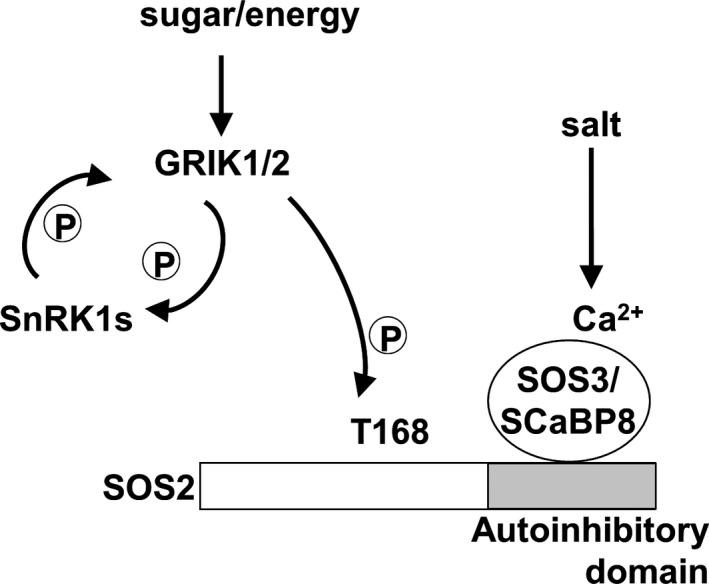
Model of the placement of GRIK kinases at the interface between sensing of the energy status and salinity stress signalling. Salt stress induces the activation of SOS2 through calcium/CBL (SOS3 and SCaBP8) binding. Sugar/energy status is transmitted through SOS2 phosphorylation by GRIK1/2, which also controls reciprocally the activity of SnRK1s.

Our data show that T168 in the activation‐loop of SOS2 is important for function of SOS2 under salt stress *in vivo* (Figure 5). Mutations at S159 and Y175 also affected salt tolerance, as reflected by root growth, although these residues were not essential for plant survival under acute salt stress (Figure 5). These two sites might be important for the local molecular conformation of SOS2, thereby exerting an effect on phosphorylation on T168, or for another unknown mechanism involving the activation‐loop such as phosphorylation by additional kinases contributing to the full activation of SOS2 under salinity. In any case, our data demonstrate that phosphorylation of T168 by GRIK1 is important for SOS2 function *in vitro* and in the yeast system. The *grik1‐2 grik2‐1* double mutant, however, was less sensitive to salt than the *sos2‐2* mutant. If GRIKs are the upstream kinases of SOS2 *in vivo*, then either the putative protein encoded by the *grik1‐2* allele works to some extent or parallel mechanisms responsible for salt resistance exist, for example another upstream kinase(s) contributing to the full activation of SOS2, perhaps by phosphorylating residues S159 and/or Y175. SOS2 is also activated by SOS3 and SCaBP8 in a Ca^2+^‐dependent manner (Halfter, 2000; Quan *et al*., 2007). GRIK‐mediated phosphorylation may affect the competence of SOS2 for Ca^2+^‐dependent activation. In that case, when salt stress induces Ca^2+^ signalling, SOS3 or SCaBP8 may activate SOS2 depending upon the sugar/energy information gating by GRIKs (Figure 8).

In summary, GRIKs can phosphorylate and activate members of the SnRK3 family in addition to their well‐known targets SnRK1s, and they play important roles not only in the sugar signalling pathway but also in the SOS pathway for salt tolerance *in vivo*.

## Experimental procedures

### Construction and site‐directed mutagenesis

Total RNA prepared from 10 days seedling of *A. thaliana* Columbia‐0 ecotype was primed with oligo(dT) and reverse‐transcribed with the Superscript II RT (Invitrogen). *GRIK1* cDNA was amplified by PCR with KOD polymerase (Takara) under the following conditions: 1 × 95°C for 5 min; 30 × (95°C for 20 sec, 55°C for 20 sec, 70°C for 2 min). Primers used to obtain a 1207‐bp fragment encoding the *GRIK1* are listed in Table S1. The PCR product was subcloned into pGEX4T1 vector between *EcoRI* and *XhoI* sites. Inserted fragments of all constructs were sequenced.

*GRIK1* cDNA was inserted into the vector p425GPD (Mumberg *et al*., 1995) as *Bam*HI/*Eco*RI for expression in yeast. Plasmids for the expression in yeast of core components of the SOS pathway have been described elsewhere (Quintero *et al*., 2002, 2011).

To introduce the point mutations into SOS2‐pGEX4T1 (Guo *et al*., 2001), primer pairs in Table S1 were used for the first PCR: 1 × 95°C for 5 min; 20 × (95°C for 20 sec, 51°C for 20 sec, 70°C for 90 sec). Using the PCR products as templates, the second amplification was performed under the following conditions: 1 × 95°C for 5 min, 30 × (95°C for 20 sec, 51°C for 20 sec, 70°C for 90 sec). The final product was cloned into pGEX4T1 (*BamHI*‐*EcoRI* sites for *SOS2*). For expression in *A. thaliana*, fragments were cloned into a binary vector (pCAMBIA1200) under the control of the CaMV 35S promoter derived from pRT105. For expression in yeast, the full‐length mutant allele *SOS2‐AAA* and the truncated version *SOS2‐AAA∆308* were subcloned as *Bam*HI/*Eco*RI fragments in p414GPD (Mumberg *et al*., 1995).

### Arabidopsis T‐DNA insertion lines

The seeds of T‐DNA insertion lines (Salk_142938 and Salk_015230) were obtained from Arabidopsis Biological Resource Center (Alonso *et al*., 2003). Homozygous insertion lines were identified with PCR following the instructions (http://signal.salk.edu/cgi-bin/tdnaexpress). The following conditions were used: 1 × 95°C for 5 min; 35 × (95°C for 20 sec, 55°C for 20 sec, 70°C for 1 min) with primers described in Table S1. The insertion sites were identified by sequencing the amplicons.

cDNAs purified from 10‐day‐old seedlings of Col‐0 plants and T‐DNA insertion lines as mentioned above were used as templates for RT‐PCR. The following conditions were used: 1 × 95°C for 5 min; 35 × (95°C for 20 sec, 58°C for 20 sec for *GRIK1* and *GRIK2*, or 54°C for 20 sec for tubulin, respectively, 70°C for 45 sec) with primers as listed in Table S1.

Northern blot analysis was performed as described in Fujii and Zhu (2009). For *GRIK1*, the cDNA fragment digested with PstI (248‐889 of ORF) was used as a probe. The mRNA of *grik1‐2* was amplified by RT‐PCR with primers given in Table S1, followed by sequencing.

### Hydroponic culture with LAK medium

For salt treatment in hydroponic culture, seeds were directly grown on hydroponics as described by Barragan *et al*. (2012). A modified Long Ashton mineral solution with 1 mm K^+^ and nominally free of Na^+^ and NH_4_^+^ (LAK medium) was used for hydroponic cultures. This medium was designed to maximize the toxicity of Na^+^ ions, while minimizing the osmotic effects of supplemental NaCl. The final composition of the LAK base solution was as follows: 1 mm KH_2_PO_4_, 2 mm Ca(NO_3_)_2_, 1 mm MgSO_4_, 30 mm H_3_BO_3_, 10 mm MnSO_4_, 1 mm ZnSO_4_, 1 mm CuSO_4_, 0.03 mm (NH_4_)_6_Mo_7_O_24_ and 100 mm Fe^2+^ as *Sequestrene* 138‐ Fe, pH 5.3. Seedlings were grown in LAK medium for 1 week followed by salt treatment at the indicated NaCl concentrations.

### Expression and purification of GST fusion proteins in *Escherichia coli*

The constructs encoding GST‐fused proteins were transformed into *E. coli* Rosetta cells (Novagen). Single colonies were grown overnight at 37°C, transferred to fresh 20 × volume of Luria‐Bertani media, and further cultured for 1 h. Recombinant protein expression was induced by 0.2 mm isopropyl beta‐d‐thiogalactopyranoside for 4 h at 37°C. The cells were harvested by centrifugation (5000 ***g***, 5 min, 4°C), and the pellets were resuspended in pre‐chilled lysis buffer (10 mm Tris pH 8.0, 150 mm NaCl, 1 mm EDTA and 100 μg ml^−1^ lysozyme), incubated on ice for 15 min. After dithiothreitol (50 mm), phenylmethanesulphonyl fluoride (1 mm) and Triton X‐100 (1.5%) were added, and the suspension was centrifuged at 30 000 ***g*** for 5 min at 4°C. Then, glutathione‐agarose beads (Sigma) were added to the supernatant, and the mixture was incubated with gentle agitation for at least 1 h at 4°C. The beads were washed six times with pre‐chilled buffer (10 mm Tris pH 8.0, 150 mm NaCl, 1 mm EDTA).

### Western blotting

Two‐week‐old Col‐0 and *grik1‐2 grik2‐1* seedlings, grown on MS media with 1% sucrose, were collected and ground in liquid nitrogen. The tissue samples were added to Laemmli sample buffer (Laemmli, 1970) and heat‐treated (10 min at 65°C). Solid material was removed by centrifugation, and samples were run in sodium dodecyl sulphate–polyacrylamide gel electrophoresis (SDS–PAGE) followed by Western blot. The blots were incubated with either anti‐phospho‐T172‐AMPK‐α antibody (Cell Signaling, MS, USA) or anti‐SnRK1.1 antibody (Agrisera, Sweden) overnight at 4°C with slow agitation. All blots were then incubated with horseradish peroxidase‐conjugated anti‐rabbit antibody (GE Healthcare, UK) for 2 h at room temperature with slow agitation. All antibody dilutions were made in TTBS‐buffer (20 mm Tris‐HCl pH 7.5, 150 mm NaCl and 0.05% Tween‐20). Signals from the blots were detected using Westernbright ECL (Advansta, CA, USA), and band strengths were evaluated with LiCor Image Studio program (LiCor, UK). Experiments were replicated with five biological samples.

### *In vitro* kinase assays

*In vitro* phosphorylation assays were performed as described previously (Fujii and Zhu, 2009), with some modification. Twenty microlitres of the reaction mixture contained 20 mm Tris (pH 7.2), 10 or 40 mm MgCl_2_, 10 μm ATP, 5 μCi [γ‐^32^P] ATP and 2 mm dithiothreitol. Reaction mixtures were incubated at 30°C for 40 min. The reaction was stopped by the addition of Laemmli's sample buffer, followed by SDS–PAGE.

### Yeast strains and media

Yeast strain YP890 (*∆ena1*::*HIS3::ena4*,* nha1*::*LEU2*,* nhx1*::*KanMX*,* PGK1*_*prom*_::*AtSOS1*::*CYC1*_*ter*_; Guo *et al*., 2004) was used to test the function of wild‐type and mutant SOS2. The yeast strain YPDahl55 (*sak1Δ*::*KanMX elm1Δ*::*KanMX tos3Δ*::*TRP1*) lacking all three upstream regulatory kinases of SNF1 (Ye *et al*., 2008) was used as the starting biological material to produce a strain suitable to test activation of SOS2 by GRIK1. The *ENA1‐ENA4* gene tandem array encoding Na^+^‐ATPases was disrupted by transformation with an *ena1*::*hisG*::*URA3*::*hisG*::*ena4* gene replacement cassette, followed by selection of uracil prototrophs and sodium‐sensitive transformants. Gene replacement was confirmed by diagnostic PCR. Next, a loss‐of‐function mutant of the *TRP1* gene marker in *tos3Δ*::*TRP1* was isolated by counter‐selection with 5‐fluoroanthranilic acid (Toyn *et al*., 2000). The resulting strain was denoted Δ3K4E. Transformation of *S. cerevisiae* was performed using a standard lithium acetate–polyethylene glycol method. Yeast cells were propagated in rich YPD medium (1% yeast extract, 2% peptone, 2% glucose). To test growth in non‐fermentable carbon sources, glucose was substituted by 3% ethanol, 2% glycerol. The ability of yeast cells to grow in salt was tested on AP medium (Rodríguez‐Navarro and Ramos, 1984). Strains were cultured overnight in liquid AP medium supplemented with 1 mm KCl. After harvest, cells were resuspended and diluted decimally in distilled water. Five‐microlitre aliquots were spotted onto AP plates supplemented with 1 mm KCl and various concentrations of NaCl, and grown for 3–4 days at 28°C.

### BiFC experiments in *Nicotiana benthamiana*

For BiFC assays, the full‐length cDNA of *GRIK1* was transferred to the pSPYCE(M) vector (Waadt *et al*., 2008) using the *Xba*I and *Sma*I sites to create a C‐terminal translational fusion of GRIK1 to the C‐terminal moiety of YFP. The N‐terminal fusion of SOS2 to the N‐terminal moiety of YFP in pSPYNE(R)173 has been described before (Waadt *et al*., 2008). All the YFP fusions were expressed under the control of the 35S promoter. The resulting plasmids were electroporated into *Agrobacterium tumefaciens* (strain GV3101). The bacterial cultures were grown at 28°C overnight and centrifuged at 15 000 ***g*** for 10 min. Pellets were resuspended with infiltration buffer (10 mm MES pH 5.6, 10 mm MgCl_2_, 0.1 mm acetosyringone) and kept at room temperature for 5 h. Cell cultures were adjusted to a final OD_600 nm_ of 0.2. Appropriate combinations of cultures were mixed with equal amounts of an Agrobacterium suspension carrying the p19 suppressor of post‐transcriptional gene silencing (Silhavy *et al*., 2002). The Agrobacterium suspensions were then infiltrated into the leaves of 3–4‐week‐old *N. benthamiana* plants as described (Marillonnet *et al*., 2005). The infiltrated plants were kept in a controlled growth chamber (16 h day/8 h night, 25°C/22°C, 60–70% relative humidity, 150 μmol m^−2^ sec^−1^ PAR) for 3 days until analysis by confocal microscopy. Images were taken with a FluoView FV1000 Confocal Microscope (Olympus) using a 488‐nm Ar/ArKr laser and 60 × objective. The Olympus FluoView 4.2 software was used to analyse the images.

### Statistics

Student's *t*‐test and one‐way anova followed by Tukey's multiple comparison test were performed for single and multiple comparisons, respectively. For normalized values, the non‐parametric binomial test was adopted with 0.5 as *a priori* probability of > 1.

## Accession numbers

GRIK1: AT3G45240; GRIK2: AT5G60550; SOS2: AT5G35410; SOS1: AT2G01980; SOS3: AT5G24270; *grik1‐2*: Salk_142938; *grik2‐1*: Salk_015230.

## Acknowledgements

The authors thank the Arabidopsis Biological Resource Center for providing the T‐DNA insertion mutants. This work was supported by the Turku Collegium for Science and Medicine and by the Academy of Finland (Projects number 259169, 263853, 271832, 292763, 307335) to HF, by National Institutes of Health (Grant R01GM059138) to JZ, and by grants BFU2015‐64671 to JMP and BIO2015‐70946‐R to FJQ from the Spanish Ministry of Economy and Competitiveness, co‐financed by FEDER, and with additional support from the SSAC grant PJ01105105 from the Rural Development Administration, Republic of Korea.

## Conflict of interest

The authors declare that there is no conflict of interest.

## Supporting information


**Figure S1.** Other phenotypes of *grik1‐2 grik2‐1* lines.

**Figure S2.** Control experiments for the *in vitro* kinase assays.

**Figure S3.** Transgenic lines with similar expression levels of SOS2‐S159A (SA), T168A (TA) and Y175A (YA) in the *sos2‐2*.

**Figure S4.** The complementation of the SNF1 upstream kinase by GRIK regarding carbon source use.

**Table S1.** Primer sequences.
