# Supplementary figures and images for: Upstream kinases of plant SnRKs are involved in salt stress tolerance
Source: Plant J. 2017 Dec 2;93(1):107–18. doi: 10.1111/tpj.13761 (PMC5814739; doi:10.1111/tpj.13761)

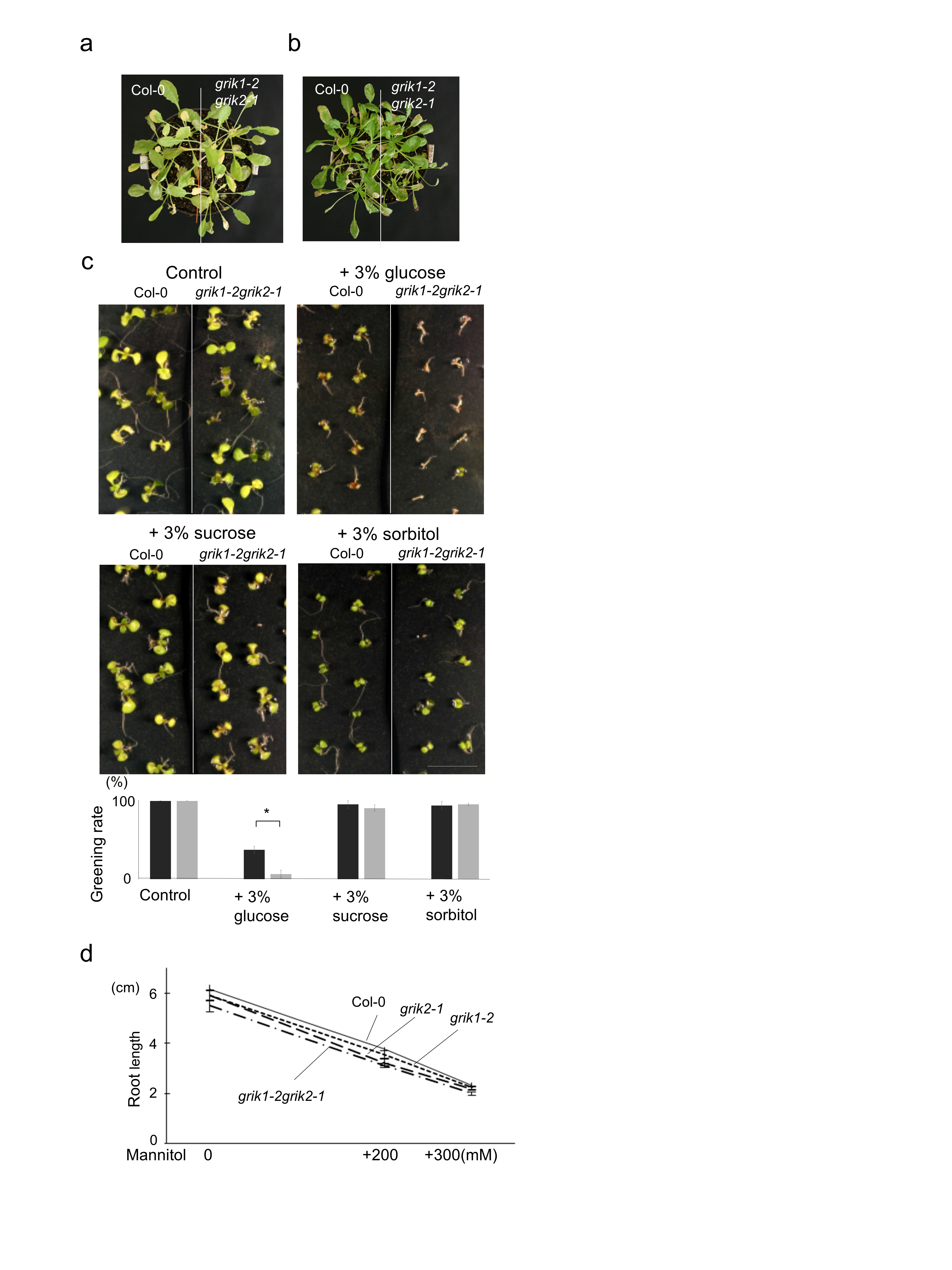

Supplement: Supplementary file 1 — Figure S1. Other phenotypes of grik1‐2 grik2‐1 lines. [file TPJ-93-107-s001.tif]

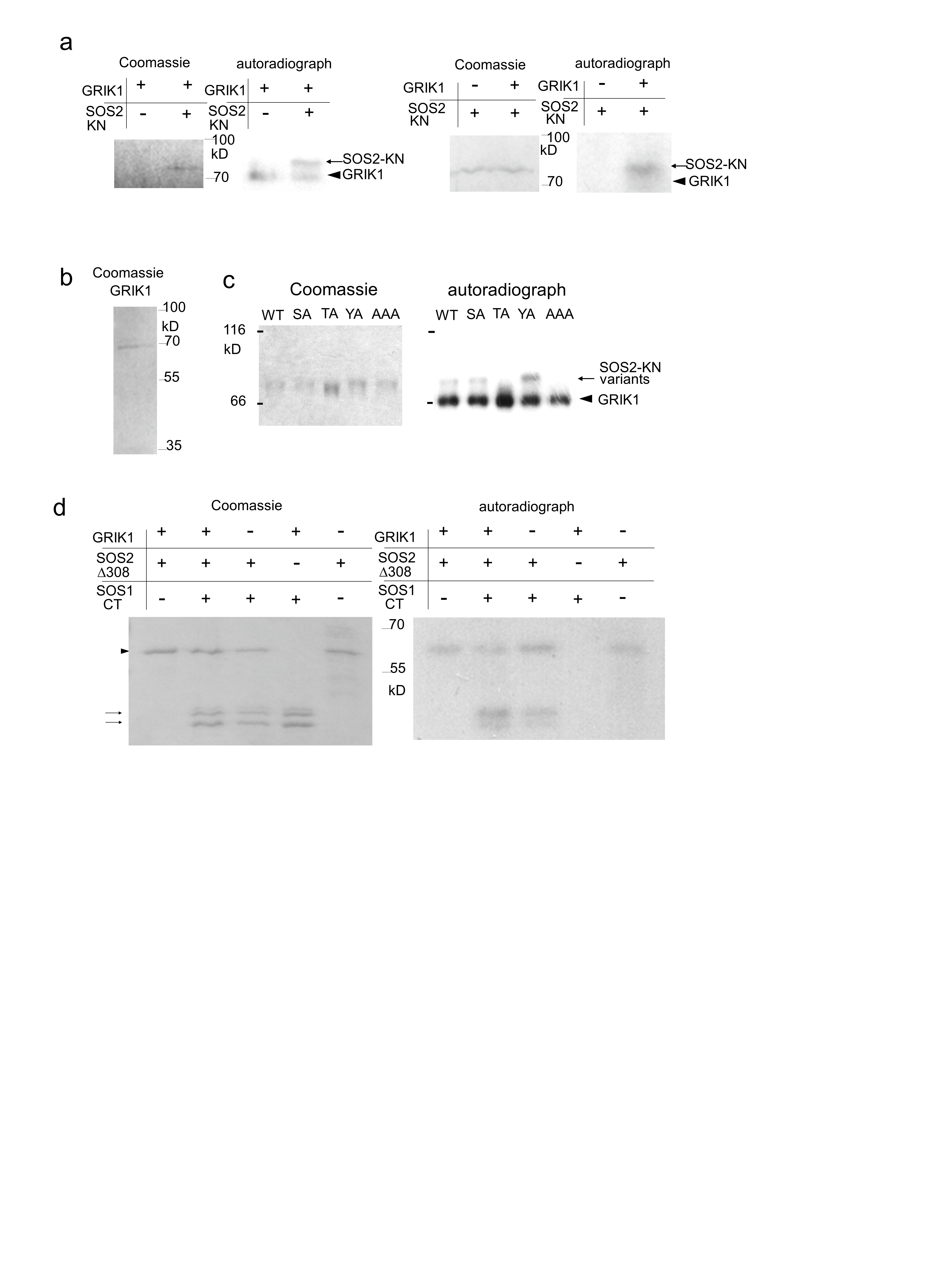

Supplement: Supplementary file 2 — Figure S2. Control experiments for the in vitro kinase assays. [file TPJ-93-107-s002.tif]

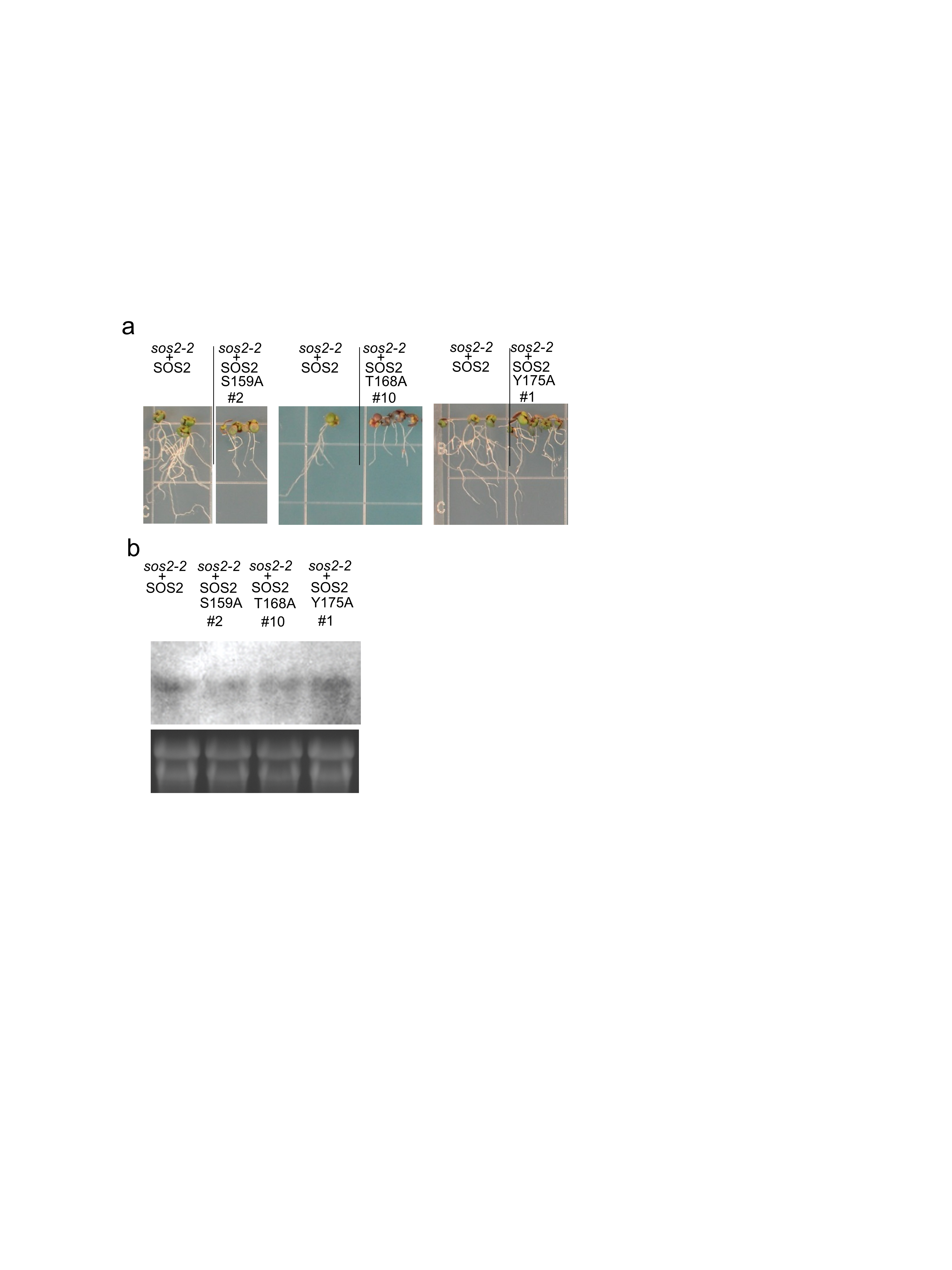

Supplement: Supplementary file 3 — Figure S3. Transgenic lines with similar expression levels of SOS2‐S159A (SA), T168A (TA) and Y175A (YA) in the sos2‐2. [file TPJ-93-107-s003.tif]

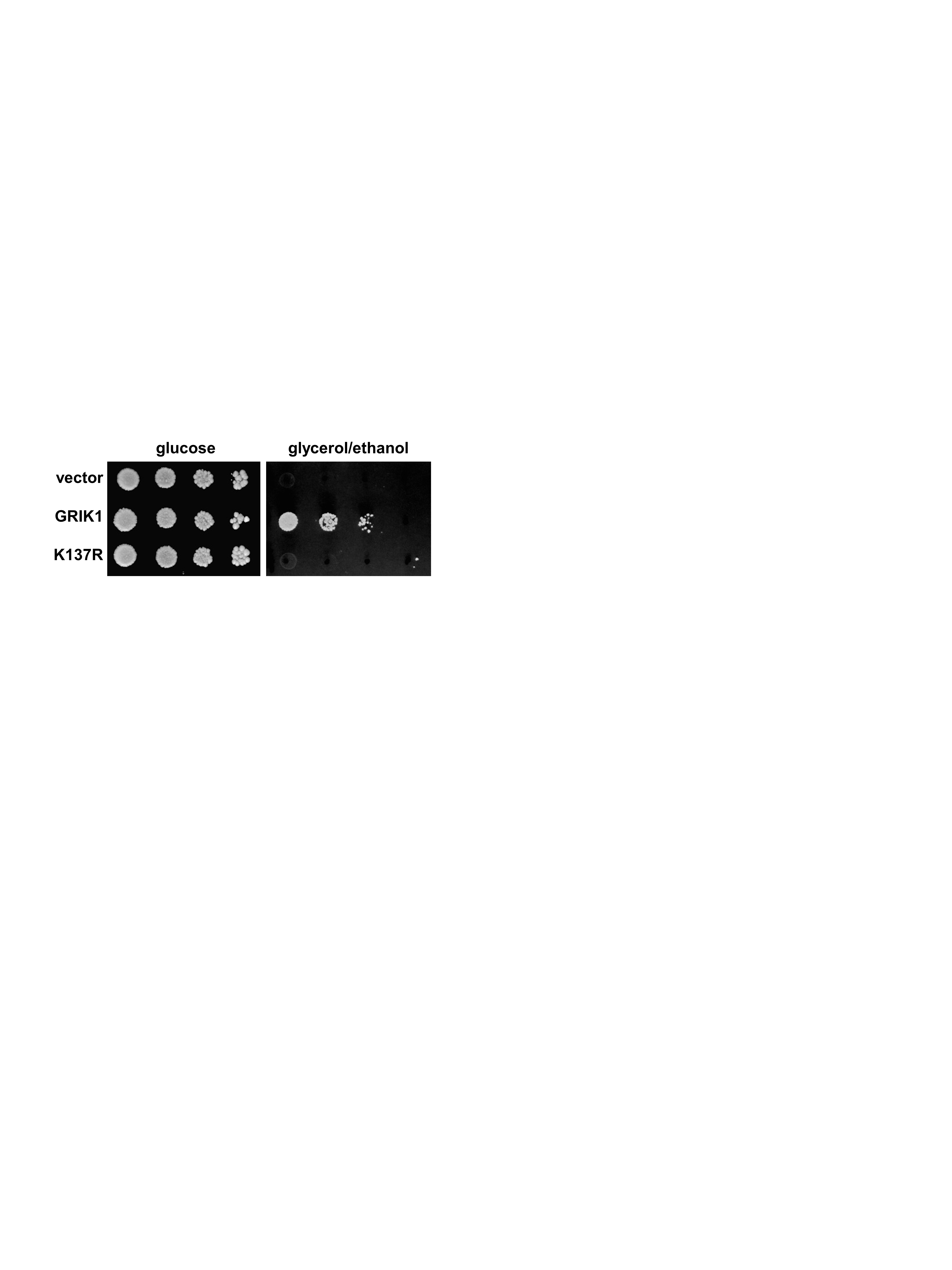

Supplement: Supplementary file 4 — Figure S4. The complementation of the SNF1 upstream kinase by GRIK regarding carbon source use. [file TPJ-93-107-s004.tif]
