# Supplementary material for: Upstream kinases of plant SnRKs are involved in salt stress tolerance
Source: Plant J. 2017 Dec 2;93(1):107–18. doi: 10.1111/tpj.13761 (PMC5814739; doi:10.1111/tpj.13761)
Supplement: Supplementary file 5 — Table S1. Primer sequences. [file TPJ-93-107-s005.docx]

**Supplemental Table 1**

Primer sequence

| GRIK1  cloning | forward for pGEX4T1 | 5'-TAGAATTCATGTTTTGTGATAGTTTTGCATTTGCCC-3' |
| --- | --- | --- |
|  | forward for p414GPD | 5'-AAGGATCCACCATGTTTTGTGATAGTTTTGCATTTGC-3' |
|  | reverse | 5'-TACTCGAGTCAGCTATGGTTTTGATCTTCTTCTTCT-3' |
| point mutation | pGEX4T1forward | 5'-GTGGTGGCGACCATCCTCCAA-3' |
|  | SOS2 S159A reverse | 5'-GCAATGCAGCTAGCCCGAAATCCGAAACCTTCAG-3' |
|  | SOS2 T168A reverse | 5'-GTTCCACACGTGGCACGCAGAAGTTCTACTCCTTCC-3' |
|  | SOS2 Y175A reverse | 5'-TGGAGCTACAGCGTTCGGAGTTCCACATGTGGTA-3' |
|  | GRIK1 K137R reverse | 5'-AAAAGCCCGGATAGCATAATGCTTGTCATCG-3' |
|  | pGEX4T1 reverse | 5'-TGTGTCAGAGGTTTTCACCGTC-3' |
|  | SOS2 S159A forward | 5'-GGATTTCGGGCTAGCTGCATTGCCTCAGGAAGGAGT-3' |
|  | SOS2 T168A forward | 5'-TCTGCGTGCCACGTGTGGAACTCCGAACTATGTAGC-3' |
|  | SOS2 Y175A forward | 5'-AACTCCGAACGCTGTAGCTCCAGAGGTACTTAGTG-3' |
|  | GRIK1 K137R forward | 5'-TATGCTATCCGGGCTTTTCACAAGTCACATT-3' |
| TDNA  line identification | GRIK1-2 LP | 5'-TGGAGTTTCGACATGTTGCGAT-3' |
|  | GRIK1-2 RP | 5'-TGGTGATCCAATTTCCAATGAGC-3' |
|  | GRIK2-1 LP | 5'-TTTTGGAGCATCCTAACATCG-3' |
|  | GRIK1-1 RP | 5'-TGAACCATTGGAGGCTAAAAGA-3' |
| to check expression | GRIK1 forward | 5'-GGTCTCCTTTGCAAAGATCCA-3' |
|  | GRIK1 reverse | 5'-AAGAATTCAAGAGTCTTCTCAATACATGACCG-3' |
|  | GRIK2 forward | 5'-GGTCTCCTTTGCAAAGATCCG-3' |
|  | GRIK1 reverse | 5'-AAGAATTCATGACCCCAAGCGAGGTC-3' |
|  | tubulin forward | 5'-TCGTGGATCACAGCAATACA-3' |
|  | tubulin reverse | 5'-CCTCCTGCACTTCCACTTCG-3' |
| BiFC | GRIK1 forward | 5’-ccgcgtggatccatgttttgtgatagttttg -3’ |
|  | GRIK1 reverse | 5’-cggcgcccggggctatggttttgatcttc -3’ |
| RT-PCR | T-DNA end forward | 5'-CATTAAAAACGTCCGCAATGTG-3' |
|  | GRIK1 1^st^ strand reverse | 5'-TCTCCCGGTACTAGTAACCAG-3' |
|  | GRIK1 4^th^ exon reverse | 5'-GAAGAACATCACCCATAGCAG-3' |
